# NF-κB repressing factor downregulates basal expression and mycobacterium tuberculosis induced IP-10 and IL-8 synthesis via interference with NF-κB in monocytes

**DOI:** 10.1186/s12929-014-0071-5

**Published:** 2014-08-19

**Authors:** Kuo-Hsiung Huang, Chun-Hua Wang, Chien-Huang Lin, Han-Pin Kuo

**Affiliations:** 1 Graduate Institute of Medical Sciences, College of Medicine, Taipei Medical University, 250 Wu-Hsing Street, Taipei 110, Taiwan; 2 Pulmonary Medicine Research Center, Chang Gung Memorial Hospital, Taipei, Taiwan; 3 Department of Thoracic Medicine, Chang Gung Memorial Hospital, 199 Tun-Hwa North Road, Taipei, Taiwan

**Keywords:** Tuberculosis, IP-10, IL-8, NF-κB repressing factor

## Abstract

**Background:**

Our previous study showed NF-κB repressing factor (NKRF) downregulates IP-10 and IL-8 synthesis in the peripheral blood mononuclear cells and alveolar macrophages of TB patients with high bacterial loads. However, the mechanism underlying the repressive effect of NKRF is not fully understood.

**Results:**

The levels of IP-10, IL-8 and NKRF were significantly up-regulated in THP-1 cells treated with heated mycobacterium tuberculosis (H. TB). NKRF inhibited NF-κB-mediated IP-10 and IL-8 synthesis and release induced by H. TB. The repressive effect of NKRF is mediated via interference with NF-κB (p65) binding and RNA polymerase II recruitment to promoter sites of IP-10 and IL-8.

**Conclusions:**

We have elucidated that direct contact with MTb induces IP-10, IL-8 and a concomitant increase in NKRF in THP-1 cells. The up-regulated NKRF serves as an endogenous repressor for IP-10 and IL-8 synthesis to hinder host from robust response to MTb infection.

## Background

Tuberculosis (TB) killed an estimated 100 million people over the last century [1]-[3]. About one third of the world’s population is infected with *Mycobacterium tuberculosis* (MTb). Most of the infected persons never develop an active disease [1]-[3] because the host immune response keeps the infection under control. After MTb infection, innate immunity initially predominates the subsequent response in the host. For the purpose to contain MTb, T lymphocytes are recruited to the lung within granulomas, which consist of activated macrophages, T lymphocytes, fibroblasts, and epitheloid cells [4]. To prevents the disease reactivation, a complex interaction between different cell populations are involved in the control of MTb infection. Specific chemokines, such as IP-10, IL-8, MIG/CXCL9 and MCP-1/CCL2 are released from monocytes, alveolar macrophages and polymorphonuclear granulocytes to recruit NK cells, γδ T lymphocytes, and αβ T lymphocytes of CD4^+^ and CD8^+^ phenotypes in sequential order into the site of MTb infection [5]-[9].

IP-10, a member of the α-chemokine subfamily, is involved in delayed type hypersensitivity [10]. It promotes Th1 responses and IFN-γ gene expression [11], and attracts monocytes and activated T lymphocytes to inflammatory foci [12]. High levels of IP-10 were detected in TB patient’s sera [13],[14] and bronchoalveolar lavage [13],[15]. Except for chemotaxis, IP-10 also contributes to the necrosis of tuberculous granulomas by inhibiting angiogenesis [16].

Enhanced IL-8 release and gene expression in macrophages or monocytes has been shown after exposure to MTb and its components [17],[18]. IL-8 gene polymorphism is associated with susceptibility to TB [19], and in HIV-infected patients [20]. IL-8 is necessary for granuloma formation [21], limits the growth of intracellular MTb, and enhances the abilities in macrophage killing MTb [22].

The induction of both IP-10 and IL-8 by MTb is NF-κB dependent [16],[23]. Our previous study showed NF-κB repressing factor (NKRF) is upregulated in the circulating monocytes and alveolar macrophages of patients with active pulmonary TB, and inhibits synthesis and release of IP-10 and IL-8 [13]. NKRF is a transcriptional silencer and is implicated in the basal silencing of specific NF-κB targeting genes, including iNOS, IFN-β and IL-8 [24]-[26]. NKRF only interacts with specific NRE (negative regulatory element) to mediate NF-κB transcriptional silencing. NKRF specific NREs are only found in certain NF-κB transcriptional genes in certain cells [24]-[26]. However, the mechanism underlying NKRF up-regulation and its silencing effect on IP-10 and IL-8 in MTb infected monocytes has not been clearly explored. In the present study, we have demonstrated that direct exposure to MTb upregulates NKRF expression in monocytes, and the repressive effect of NKRF on IP-10 and IL-8 synthesis might be via interfering with NF-κB (P65) binding and RNA polymerase II recruitment to their promoter sites.

## Methods

### Cell preparation and culture

THP-1 cells purchased from the ATCC (TIB202) were grown in suspension in T-150 tissue culture flasks in RPMI 1640 (GIBCO, Grand Island, NY, USA) supplemented with 10% fetal calf serum (FCS, Flow Laboratories, Paisley, Scotland, UK). Cells (1 × 10^6^ cells/ml) were pretreated with or without NF-κB specific inhibitor Helenalin (Merck KGaA, Darmstadt, Germany) half hour before incubation with or without heated TB bacilli (H37-RA) (H. TB) (DIFCO) for various time points (6, 24, 72 hrs). The culture supernatant was collected and frozen at −70°C before analysis for assay of IP-10 and IL-8 by ELISA with commercial ELISA kits (R&D Systems, Minneapolis, MN). The levels of cytokine secretion could vary among different passages of the target cells, *e.g*., THP-1 cells. In each experimental study, the same passage of cultured cells was used to minimize the variation. The control group from the same passage was used to justify a similar response as that in different passage of cultured cells.

### Quantitative real-time PCR (qPCR)

Total RNA was isolated from cells using TRIzol reagent (Invitrogen, Grand Island, NY) according to the manufacturer’s instruction. cDNA was reversely-transcribed from isolated RNA by incubating 200 ng of DNase-treated RNA with the first-strand synthesis kit (Advanced Biotechnologies). qPCR was performed in a LightCycler 2.0 System (Roche Applied Science) using LightCycler DNA Master SYBR Green I (Roche Applied Science). Samples were denatured at 95°C for 10 min, followed by 45 cycles of annealing and extension at 95°C for 15 s, 60°C for 5 s, and 72°C for 10 s. Melting curves were obtained at the end of amplification by cooling the samples to 65°C for 15 s, followed by further cooling to 40°C for 30 s.

Data were analyzed by standard curve method of relative quantification using the LightCycler analysis software.

### Quantification of NF-κB p65 DNA-binding activity (TransAM assay)

To determine the alteration of NF-κB activity by MTb in THP-1 cells, the level of NF-κB subunits p50, p52, p65, C-Rel and RelB activity was measured using the NF-κB TransAM kit (Active Motif) according to the manufacturer’s instructions. Briefly, cells nuclear extraction were prepared by using the Nuclear Extract Kit (Active Motif) and protein concentrations were measured using the Bradford assay (Bio-Rad). Lysates (10 μg total proteins) were incubated in ELISA wells coated with the NF-κB consensus site (5′-GGGACTTTCC -3′) recognized by active NF-κB subunits p50, p52, p65, C-Rel and RelB, then they were detected using a specific antibody, followed by a secondary antibody conjugated to peroxidase.

### Immunostaining and confocal microscopic analysis

THP-1 cells treated with or without H. TB were spun down on slide then fixed in methanol at −20°C for 5 min. The cells were then blocked with 1% BSA/PBS at room temperature for 30 min and incubated with the rabbit anti human NKRF Ab at room temperature for 1 hr. After washing, the cells were incubated with a Cy3-conjugated anti-rabbit Ab (Chemicon International) and incubated with Hoechst dye (Sigma-Aldrich). After washing and air-drying, the cells were mounted with anti-fade mounting medium (Dako Cytomation). Images were acquired with a confocal laser-scanning microscope (Leica) and analyzed by Metamorph Image Analysis (Universal Imaging).

### Western blot analysis

Total cellular proteins were extracted from THP-1 cells by freeze-thawing samples in Reporter lysis buffer (Promega). Proteins were subjected to 7.5% SDS-PAGE and blotted onto nitrocellulose filters. NKRF was detected with β-actin (sigma) and an alkaline phosphatase-conjugated anti-mouse secondary Ab (1/100,000 dilution; Calbiochem) or specific anti-NKRF Ab and an alkaline phosphatase-conjugated anti-rabbit secondary Ab (1/10,000 dilution; Calbiochem). Blots were incubated with ECL solution (LumiGLO; Amersham Bioscience). Images were acquired and analyzed using G: BOX (Syngene).

### Transfection of siRNA and plasmids

To knockdown NKRF expression, Si-RNA (Si-Scramble and Si-NKRF) were introduced into THP-1 cells. Plasmid DNA (p-CMV and p-CMV-NKRF) were introduced into THP-1 cells to evaluate whether NKRF overexpression can regulate the release of IP-10 and IL-8. The transfection of THP-1 cells (1 × 10^6^ cells/ml) was performed by lipofectamine 2000 kit (Invitrogen, Grand Island, NY). We diluted 20 pmol siRNA oligomer (or 1 μg plasmid DNA) in 50 μl Opti-MEM medium (Invitrogen, Grand Island, NY) without serum to mix gently. Then, 1 μl Lipofectamine 2000 in 50 μl Opti- MEM medium was diluted to mix gently and incubate for 5 minutes at room temperature. The diluted oligomer and diluted Lipofectamine 2000 were mixed and incubated for 20 minutes at room temperature. We added the oligomer (DNA)- Lipofectamine 2000 complexes to each well containing 0.5 ml cells and medium. The cells were incubated at 37°C in a CO_2_ incubator for 6 hrs. After transfection, cells were incubated in complete medium for 48 hrs (for si-RNA) or 24 hrs (for plasmid DNA). The transfection efficiency in THP-1 cells was 53.4% for siRNA and 56.3% for plasmid DNA. The supernatant of cell after 6 hrs culture was collected for ELISA. The protein of transfected THP-1 cells was harvested for Western blot analysis.

### Chromatin immunoprecipitation (ChIP) assay

ChIP assays were preformed as described previously [13],[27]. After stimulation, protein-DNA complexes were cross-linked at 37°C for 10 min by formaldehyde (1% final concentration). Twenty μl (1%) of ChIP dilution solution was kept as input control. The remained diluted solution was precleared by incubating with 80 μl of salmon sperm DNA/protein A-agarose-50% slurry for 30 min at 4°C on a rotator. After centrifuge, 900 μl of the supernatant was immunoprecipitated at 4°C overnight on a rotator by using Abs specific for NKRF (5 μg), IgG (Santa Cruz Biotechnology) followed by incubation for 1 hr at 4°C with 60 μl of salmon sperm DNA/protein A-agarose-50% slurry. Protein-bound immunoprecipitated DNA (IP-DNA) was sequentially washed with low-salt or high-salt immune complex wash buffers. Immune complexes were eluted twice by adding 250 μl of elution buffer (1% SDS/0.1 M NaHCO3). DNA-protein cross-links were reversed by incubation for 4 hrs at 65°C in 200 mM NaCl/1% SDS, and proteins were digested by incubation for 1 hr at 45°C with 70 μg/ml proteinase K (Sigma-Aldrich). DNA was isolated with phenol/chloroform, precipitated with ethanol/0.3 M NaHCOOH/20 μg of glycogen and was resuspended in 50 μl of nuclease- free water. qPCR was performed with 7 μl of DNA sample for quantification.

### Statistical analysis

Data were expressed as mean ± SE. The data were analyzed with Student’s *t* test for paired or unpaired data. For data with uneven variation, the Mann–Whitney U test or Wilcoxon’s signed ranks test was used for unpaired or paired data, respectively. Statistical significance of results was determined using prism4 software. A value of p < 0.05 was considered statistically significant.

## Results

### Increased mRNA expression and protein release of IP-10 and IL-8 in H. TB treated THP-1 cells

**Figure 1:**
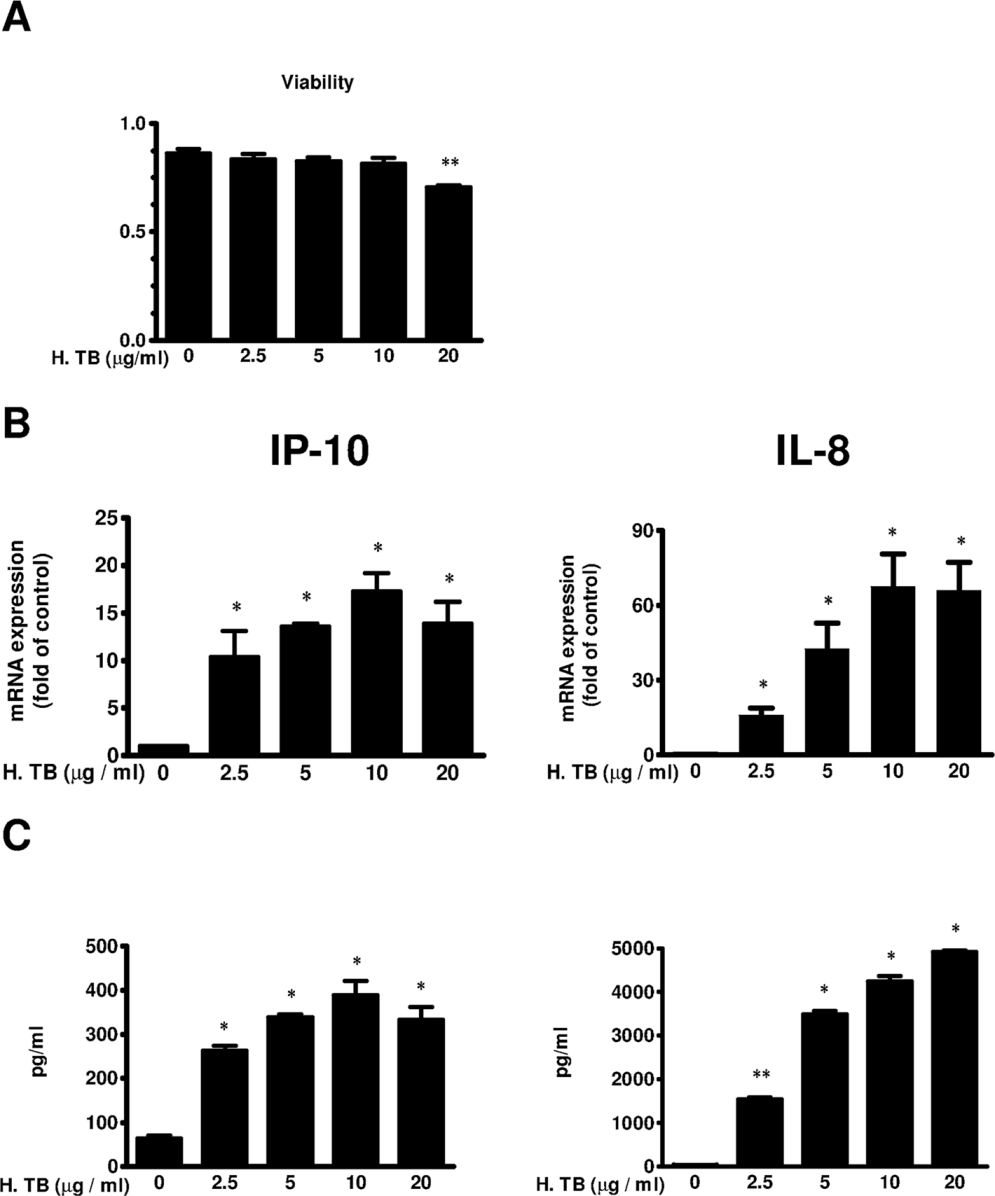
**The activation of IP-10 and IL-8 in THP-1 cells treated with H. TB. (A)** MTT cytotoxicity assay shows the cell viability of THP-1 cells when treated with H. TB at variable concentrations (2.5, 5, 10, 20 μg/ml) for 6 hours. The cell viability decreased when THP-1 cells treated with H. TB at the concentration of 20 μg/ml (n = 5) compared with control. **(B)** The expression of IP-10 (Left panel) and IL-8 (Right panel) mRNA in THP-1 (n = 5) cells was concentration-dependently increased when treated with H. TB for 2 hours, reaching the maximum at 10 μg/ml. **(C)** The release of IP-10 and IL-8 proteins by THP-1 cells (n = 5) was concentration-dependently increased when treated with H. TB for 6 hours, reaching the maximum at 10 μg/ml. Data are means ± SE. *p < 0.05, **p < 0.01 compare with the vehicle control.

After treatment with or without H. TB, THP-1 cells were harvested for q-PCR and the supernatant was collected for ELISA. The expression of mRNA and NF-κB subunit activation were observed at 2 hour, while protein expression and ChIP assay were observed at 6 hour and 1 hour, respectively throughout the study. The viability determined by MTT assay was significantly decreased when THP-1 cells treated with 20 μg/ml H. TB (81.7 ± 1.0% of control) (Figure 1A). Among non-cytotoxic concentrations (H. TB 2.5, 5, 10 μg/ml), 10 μg/ml induced the maximal and 5 μg/ml induced the submaximal cytokines secretion responses. We therefore defined H. TB 10 μg/ml as the highest non-cytotoxic concentration and 5 μg/ml as submaximal concentration in this study. H. TB induced increases in the levels of mRNA and protein of IP-10 and IL-8 in a concentration-dependent manner (Figures 1B, C). When treatment with H. TB for 2 hrs, the mRNA levels of IP-10 significantly increased at 5 μg/ml of H. TB (n = 5), reaching the maximum at 10 μg/ml (n = 5) (Figure 1B). The levels of IL-8 mRNA significantly increased at 2.5 μg/ml of H. TB (n = 5), reaching the maximum at 10 μg/ml (n = 5) (Figure 1B), when compare to the vehicle controls (n = 5, P < 0.05 respectively). When treatment with H. TB for 6 hrs, the protein of IP-10 significantly increased at 2.5 μg/ml of H. TB, reaching the maximum at 10 μg/ml when compared with vehicle control (Figure 1C). The protein of IL-8 also significantly increased at 2.5 μg/ml of H. TB, reaching the maximum at 20 μg/ml when compared with vehicle controls (Figure 1C).

### Increased NF-κB subunits, and the role of NF-κB inhibitor in the release of IP-10 and IL-8 in H. TB treated THP-1 cells

**Figure 2:**
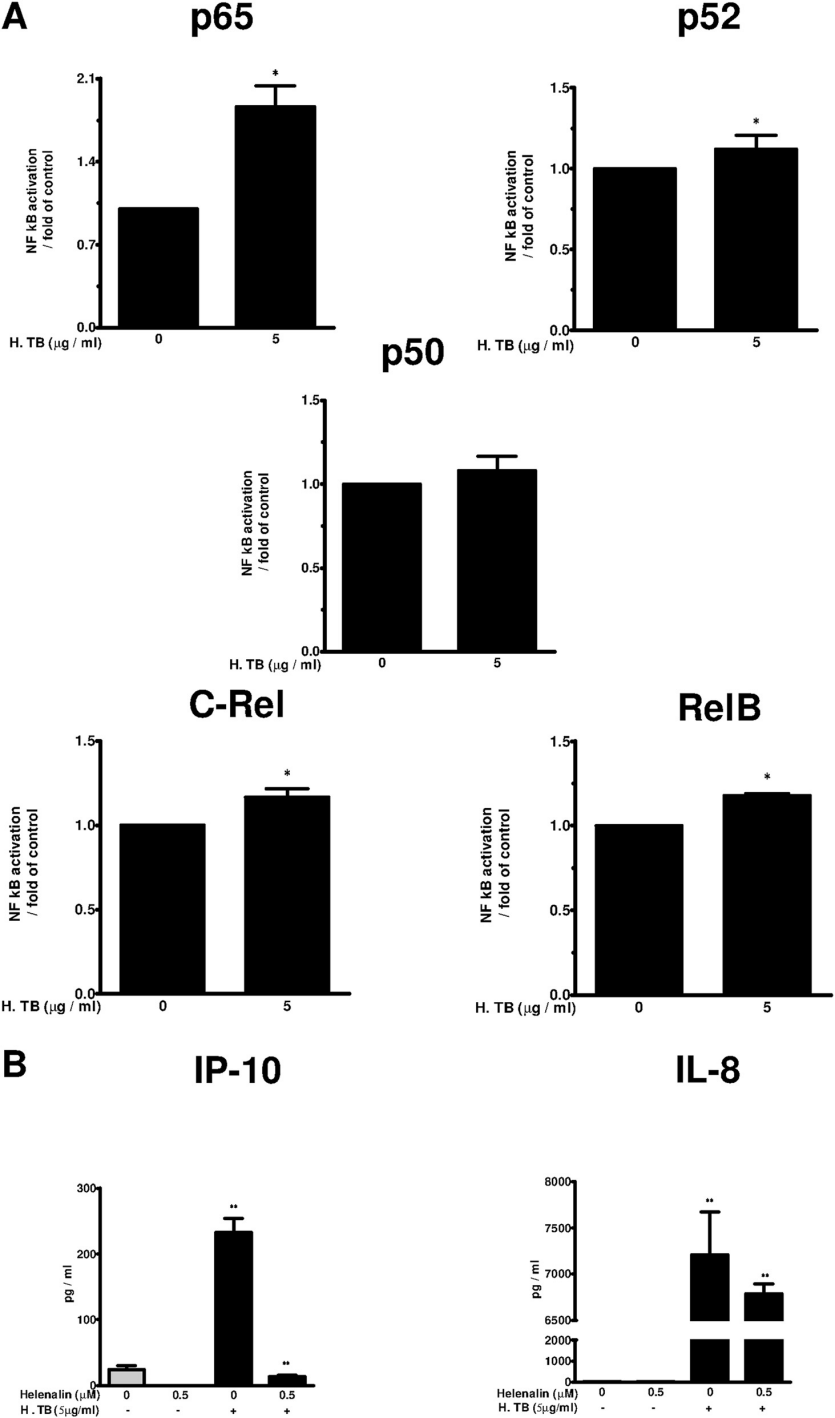
**Increased NF-κB subunits, and the role of Helenalin in the release of IP-10 and IL-8. (A)** TransAM assays to measure the NF-κB subunits p65, p52, p50, C-Rel and RelB activities reveals an increase of p65, p52, C-Rel and RelB in THP-1 cells treated with 5 μg/ml H. TB at 2 hour (n = 5). **(B)** The release of IP-10 (left panel) and of IL-8 (right panel) in THP-1 cells (n = 5) treated with H. TB for 6 hours was significantly inhibited by a NF-κB specific inhibitor Helenalin (0.5 μM). Data are means ± SE. *p < 0.05, **p < 0.01 compared with corresponding vehicle control.

This study was designed to examine the modulatory role of NKRF in regulation of IP-10 and IL-8. Therefore, a submaximal concentration at 5 μg/ml was used to examine the mechanisms for NKRF implicated in suppression of IP-10 and IL-8 gene activation via an interference with NF-κB p65. TransAM assay in THP-1 cells stimulated with H. TB (5 μg/ml) for 2 hrs revealed an increase in NF-κB subunits p65, p52, p50, C-Rel and RelB, respectively (p < 0.05, n = 5) compare to vehicle control (Figure 2A). THP-1 cells pretreated with a specific NF-κB inhibitor, Helenalin (0.5 μM), suppressed H. TB induced IP-10 and IL-8 release compared with those of control (Figure 2B), indicating the release of IP-10 and IL-8 is mediated via NF-κB. There was no significant cytotoxicity induced by 0.5 μM Helenalin (cell viability >96%, data not shown).

### Expression of NKRF in THP-1 cells

**Figure 3:**
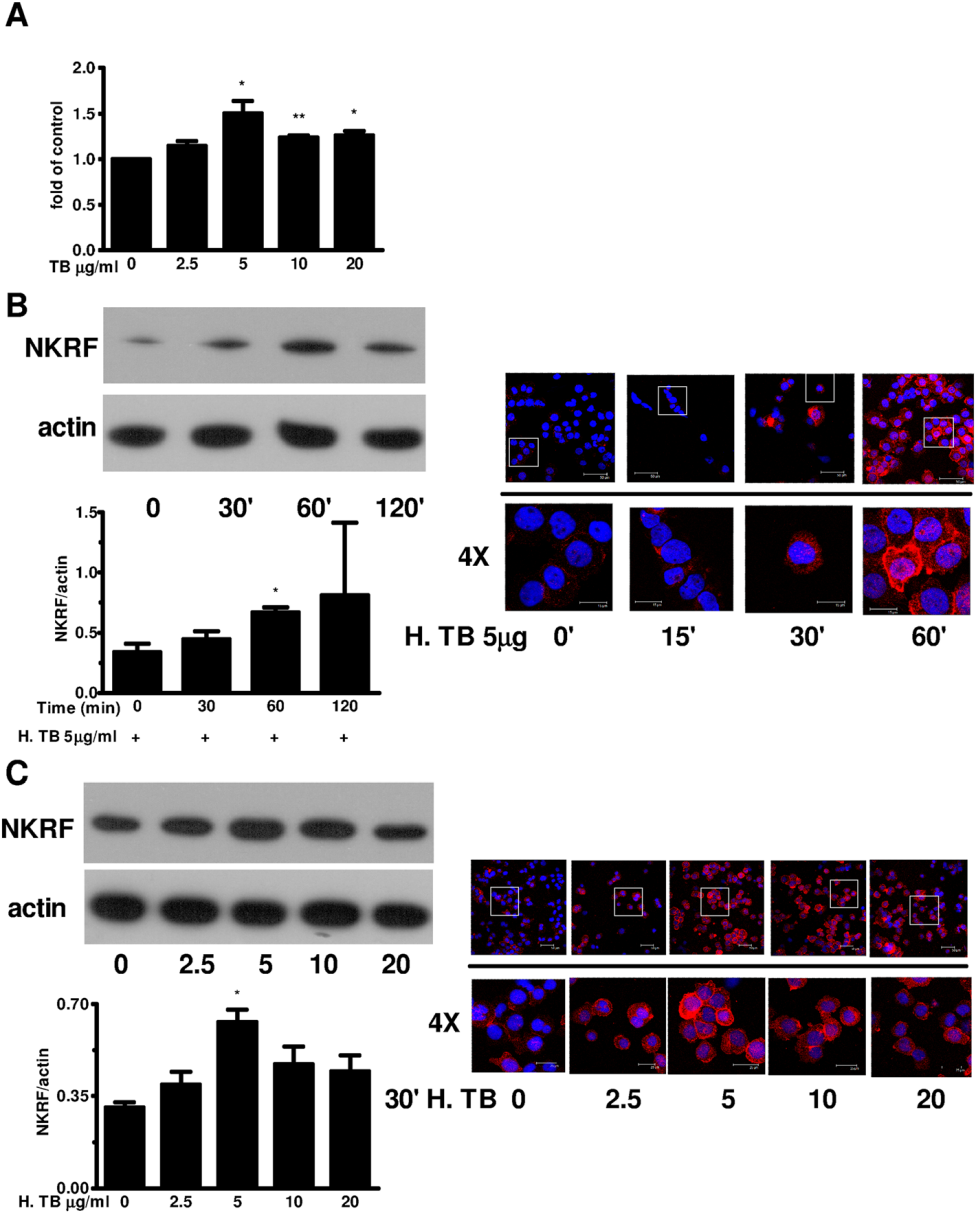
**Expression of NKRF in THP-1 cells treated with H. TB. (A)** Treatment of H. TB for 2 hours increased the expression of NKRF mRNA in THP-1 cells (n = 5) significantly at the concentration of 5, 10 and 20 μg/ml. **(B)** Westen blotting (left panel) and confocal image analysis (right panel) (n = 5) of THP-1 cells treated with H. TB at the concentration of 5 μg/ml revealed a time-dependent increase in NKRF expression. **(C)** The Western blotting (left panel) and confocal image analysis (right panel) (n = 5) for H. TB induced NKRF expression revealed a maximal response at concentration of 5 μg/ml, when THP-1 cells were treated with H. TB (2.5, 5, 10, and 20 μg/ml) for 30 minutes. Data are means ± SE. *p < 0.05, **p < 0.01 compared with control.

Quantification by quantitative RT-PCR demonstrated that NKRF mRNA expression in THP-1 cells increased when treated with H. TB (2.5, 5, 10, 20 μg/ml) for 2 hrs compared with control (Figure 3A). Western Blot analysis (Figure 3B left panel) and confocal microscopic analysis (Figure 3B right panel) revealed a time-dependent increase of NKRF protein in THP-1 cells. The Western blotting (left panel) and confocal image analysis (right panel) for H. TB induced NKRF expression revealed a maximal response at concentration of 5 μg/ml, when THP-1 cells were treated with H. TB (2.5, 5, 10, and 20 μg/ml) for 30 minutes.

### NKRF binds the IP-10 and IL-8 promoter sites in H. TB treated THP-1 cells

**Figure 4:**
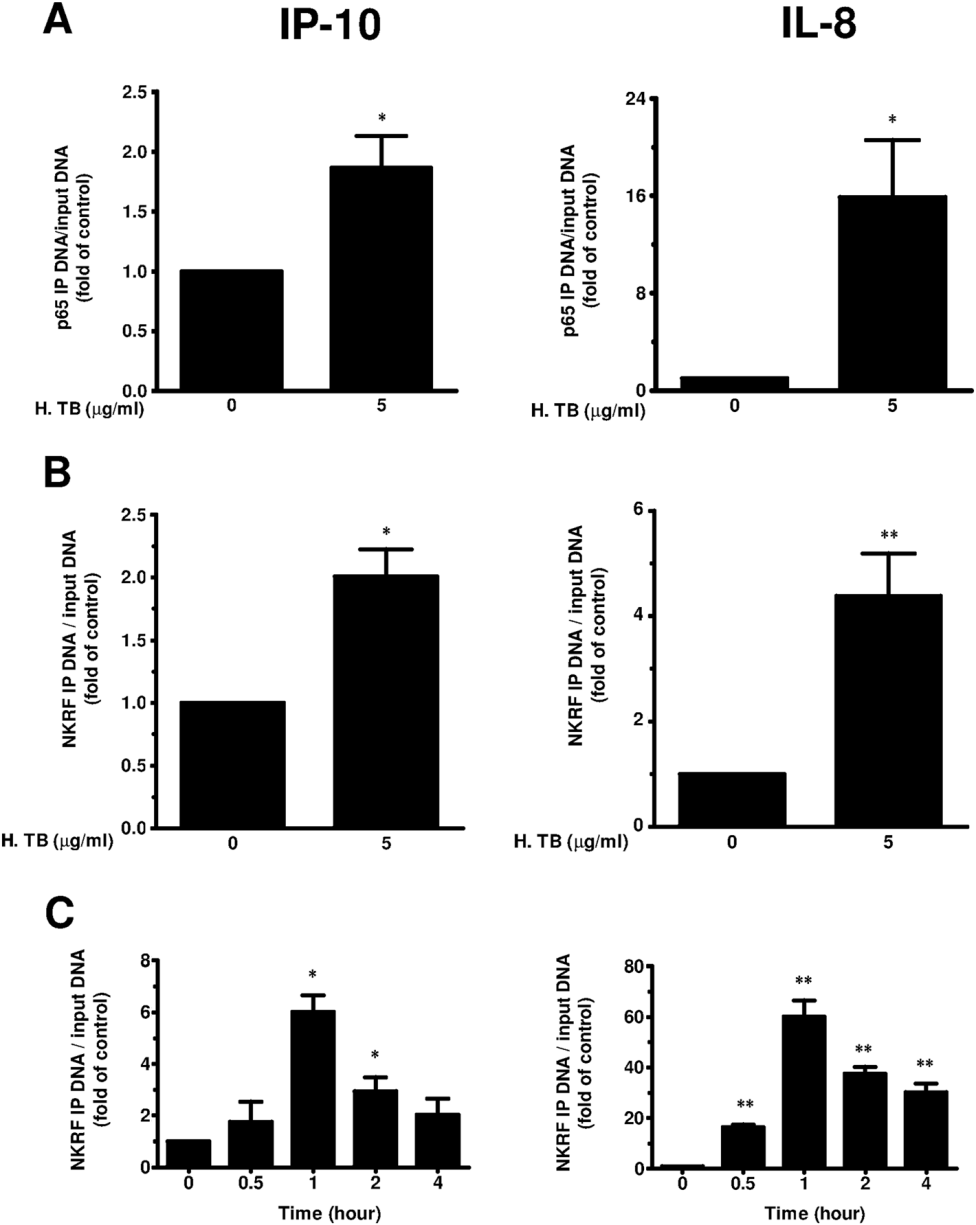
**p65 and NKRF binding to IP-10 and IL-8 promoter sites by ChIP assays. (A)** The ChIP assays showed a higher binding of p65 to IP-10 (left panel) and IL-8 (right panel) promoter sites in THP-1 cells treated with H. TB (5 μg/ml). **(B)** The ChIP assays showed a higher binding of NKRF to IP-10 (left panel) and IL-8 (right panel) promoter sites in THP-1 cells treated with H. TB (5 μg/ml) **(C)** H. TB (5 μg/ml) induced NKRF binding to IP-10 (left panel) and IL-8 (right panel) promoter sites in THP-1 cells is time-dependent. Data are means ± SE. *p < 0.05, **p < 0.01 compared with control.

By binding to the NRE in the IL-8 promoter, NKRF showed suppression of its basal transcription [28]. As shown in our previous study, there is a specific NRE sequence in the promoter site of IP-10 (Table 1). To this end, ChIP assay analysis with the antibody specific for NKRF was used to study whether NKRF would bind to the NRE sequence in the promoter sites of IP-10 or IL-8. The amount of IP-DNA was determined by RT-qPCR using primer pairs amplifying a region around the NRE site in the IP-10 or IL-8 promoter (Table 2). To demonstrate the site specificity of the assay, a primer pair amplifying an irrelevant site around the 3′-UTR was also used. IgG controls were used to demonstrate the specificity of the antibody. In the vehicle control, a low but consistently detected enrichment of IP-DNA over background (the IgG control) was observed. In H. TB treated THP-1 cells, the amount of p65 IP-DNA at IP-10 and IL-8 promoters was significantly higher than those of control (Figure 4A). In contrast, there was no significant change in the IP-DNA when the primers for 3′-UTR were used (data not shown). H. TB also induced increased occupancy of NKRF to the promoters at IP-10 and IL-8 in THP-1 cells (Figure 4B). The highest increase was found in 1 hr (Figure 4C).

**Table 1:** Sequence comparison of the NRE sites in IP-10 and IL-8 promoters

**Construct gene**	**NRE sequence**
**IP-10 promoter**	**(−503) AACTCCTGAGC (−493)**
**IL-8 promoter**	**(−1415) AATTCCTCTGA (−1405)**

NRE = negative response element; IP-10 = interferon gamma induced protein 10 kd; IL-8 = interleukin-8.

**Table 2:** Transcript and sequence of each primer used in real time RT-PCR and ChIP assays

**Transcript**	**Sequence**
**For RT-PCR**	**Primer**
IP-10	F: 5′-AGTTAGCAAGGAAAGGTCT-3′
	R: 5′-ACATTATAGTGCCAGGT-3′
IL-8	F: 5′-AGATCTGAAGTGTGATGACTCAGG-3′
	R: 5′-GAAGCTTGTGTGCTCTGCTGTCTC-3′
NKRF	F: 5′-AGAAAGATGGGTTGGACT-3′
	R: 5′-CTGTGTGGCTCTCGGA-3′
GAPDH	F: 5′-TTCCAGGAGCGAGATCCCT-3′
	R: 5′-CACCCATGACGAACATGGG-3′
**For ChIP assays**	**Primer**
NKRF promoter,	F: 5′-AGGTTCAAGCAGTTTTCC-3′
	R: 5′-CTGTAATCTCAGCACTTTGG-3′
IP-10 promoter	F: 5′-AGGCTGGTCTCAAACT-3′
	R: 5′-CCTCCCACATCCAATTACT-3′
IL-8 promoter	F: 5′-GGGCCATCAGTTGCAAATC-3′
	R: 5′-TTCCTTCCGGTGGTTTCTTC-3′
IP-10 3′-untranslated region (3′-UTR)	F: 5′- TTGAGTTATAATTACTTAT-3′
	R: 5′-TGAAAAGAAGGGTGAGAAGAG-3′
IL-8 3′-untranslated region (3′-UTR),	F: 5′-AGGTTCAAGCAGTTTTCC-3′
	R: 5′-CTGTAATCTCAGCACTTTGG-3′

F = forward; R = reverse; IP-10 = Interferon gamma induced protein 10 kd; IL-8 = interleukin-8; NKRF = NF-κB repressing factor; ChIP = chromatin immunoprecipitation.

### NKRF inhibits H. TB induced release of IP-10 and IL-8 in THP-1 cells

**Figure 5:**
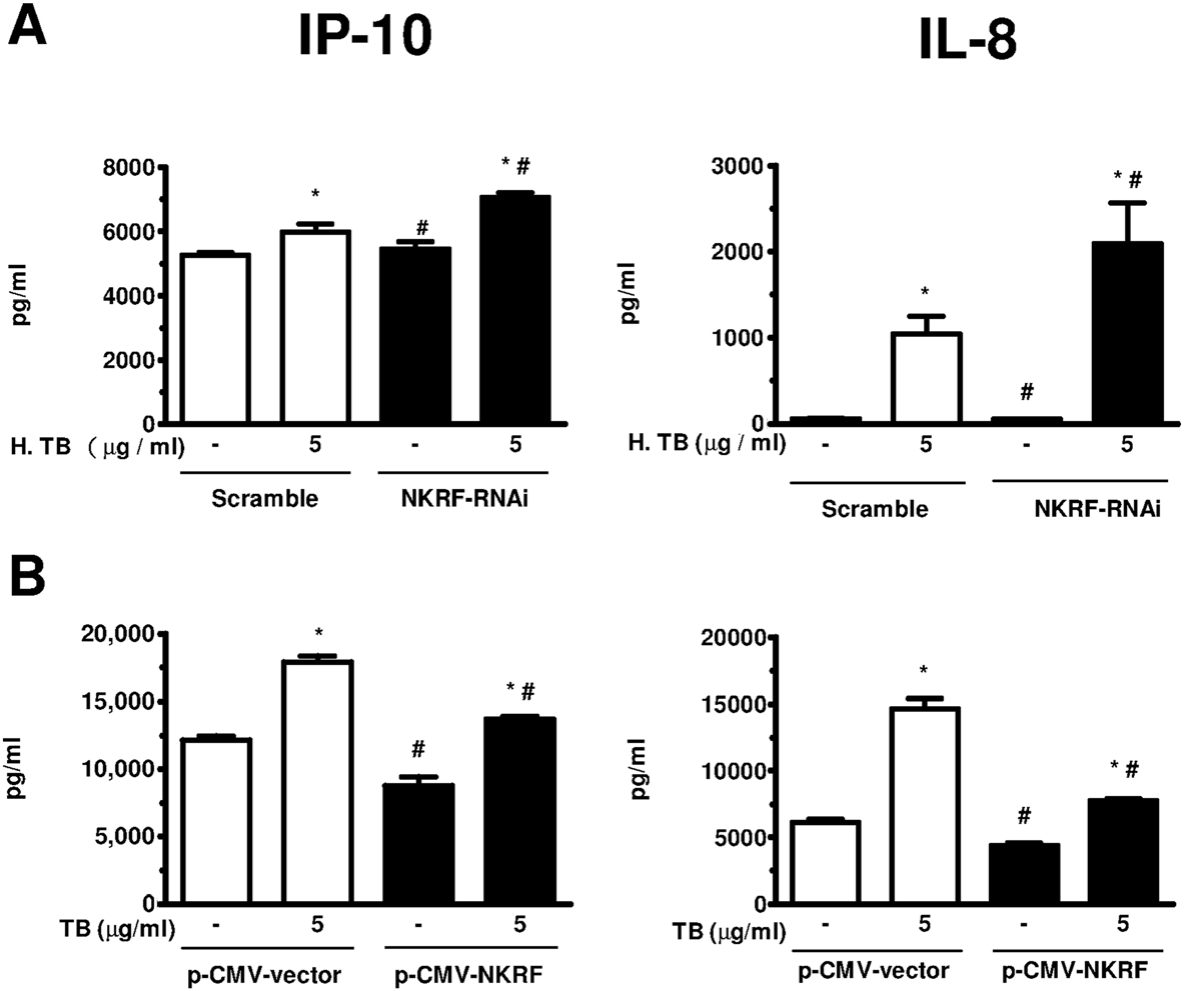
**Inhibition of NKRF increased the release of IP-10 and IL-8. (A)** THP-1 cells transfected with siRNA targeting NKRF (NKRF-RNAi) for 48 hours significantly enhanced the release of IP-10 (left panel) and IL-8 (right panel) proteins with or without H. TB (5 μg/ml) stimulation, when compared with corresponding scramble control (Scramble). **(B)** THP-1 cells transfected with NKRF plasmid (p-CMV-NKRF) for 24 hours significantly decreased the protein levels of IP-10 (left panel) and IL-8 (right panel) in THP-1 cells with or without H. TB (5 μg/ml) stimulation, when compared with corresponding vector control (p-CMV-vector). Data are means ± SE. *p < 0.05 compared with the corresponding group without H. TB treatment; #p < 0.05 compared with the corresponding Scramble or vector control group.

We conducted *in vitro* experiments of NKRF knockdown by transfection with siRNA targeting NKRF (NKRF-RNAi) or *non-targeting siRNA (scramble RNA)* in THP-1 cells. Transfection with NKRF-RNAi for 48 hrs significantly decreased the level of NKRF mRNA compared with *scramble* (55.2 ± 8.0% of scramble control, n = 5, p *<* 0.05 data not shown). MTT assays showed similar cellular viability between NKRF-RNAi and scramble transfected cells (96.6 ± 3.0% of control, n = 5, data not shown). Cells after transfection were then treated with or without H. TB for 6 hrs, and the supernatants were collected for ELISA assay. THP-1 cells transfected with NKRF-RNAi released a greater levels of IP-10 and IL-8 proteins with or without H. TB (5 μg/ml) treatment when compared with those transfected with *scramble* (Figure 5A). These data indicate that endogenous NKRF suppresses basal and H. TB induced IP-10 and IL-8 production in THP-1 cells.

**Figure 6:**
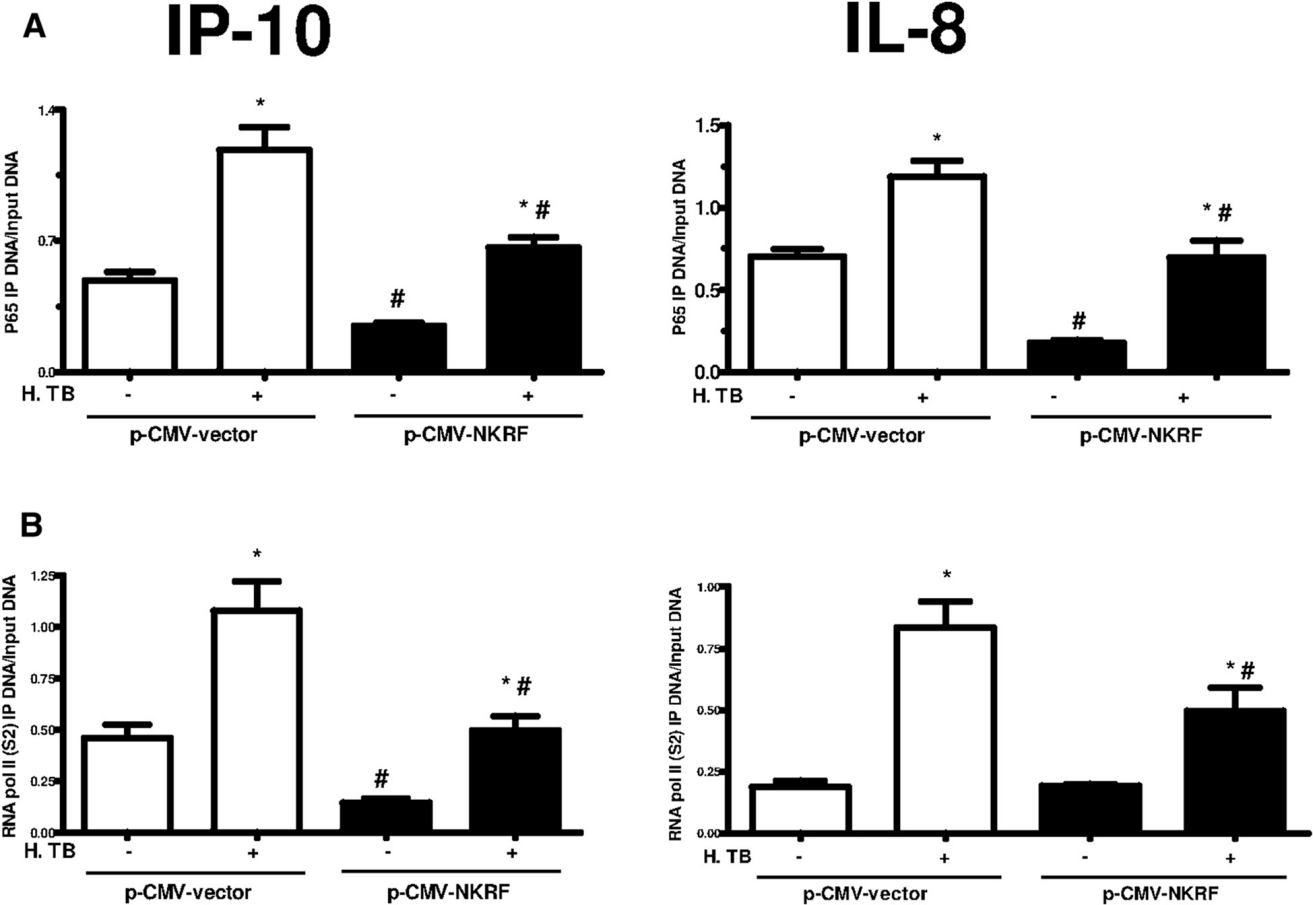
**NKRF overexpression can inhibit H. TB induced IP-10 and IL-8 release in THP-1 cells.** THP-1 cells were transfected with NKRF plasmid (p-CMV-NKRF) or control vector (p-CMV-vector) for 24 hours, and then incubated with H. TB (5 μg/ml) for 1 hr. ChIP assays were performed by using p65 or RNA pol II antibodies. The IP-DNA was quantified by q-PCR with primer pairs specific to IP-10 and IL-8 promoter sites. Values were normalized by input DNA. The ChIP assays showed a significantly decreased in p65 **(A)** and RNA pol II **(B)** binding to IP-10 (left panel) and IL-8 (right panel) promoter sites in NKRF overexpress THP-1 cells treated with or without H. TB stimulation. Data are means ± SE. *p < 0.01, compared with the group without H. TB treatment; #p < 0.05 compared with vector control group.

To delineate the mechanism underlying the up-regulation of NKRF by H. TB, THP-1 cells were transfected with plasmid DNA (p-CMV-vector and p-CMV-NKRF) for 24 hrs, then exposed to H. TB. Lower protein levels of IP-10 and IL-8 were produced by p-CMV-NKRF transfected THP-1 cells than p-CMV-vector transfected cells when exposed to 5 μg/ml of H. TB (Figure 5B). The ChIP assays revealed overexpressions of NKRF by p-CMV-NKRF transfection inhibited p65 binding to the promoter sites of IP-10 and IL-8 at basal levels or after H. TB treatment (5 μg/ml) when compared with p-CMV-vector transfection (Figure 6A). Treatment with H. TB induced recruitment of RNA polymerase II to the promoter sites of IP-10 and IL-8 when compared with vehicle controls (Figure 6B). Transfection with p-CMV-NKRF significantly attenuated H. TB induced recruitment of RNA polymerase II to either IP-10 or IL-8 promoter sites (Figure 6B). These observations suggest that the intracellular NKRF regulates the production of IP-10 and IL-8 via an inhibition of p65 binding and RNA polymerase II recruitment to both IP-10 and IL-8 promoter sites.

## Discussion

The present study has demonstrated that direct exposure to Mycobacterium TB induces increased mRNA synthesis and release of IP-10 and IL-8 proteins, as well as a concomitant upregulation of NKRF mRNA and proteins in mononuclear cells. The ChIP assay revealed that increased NKRF nuclear translocation was associated with an increased occupancy at the promoter sites of IP-10 and IL-8. Over there, NKRF hindered NF-κB (p65) from binding to the promoter sites, and then prevented RNA polymerase II recruitment resulting in a decrease in mRNA and protein synthesis of IP-10 and IL-8. Knockdown of NKRF by siRNA augmented H. TB induced protein release of both IP-10 and IL-8. Furthermore, NKRF overexpression suppressed H. TB induced release of IP-10 and IL-8 proteins. Our results illustrate that NKRF can restrain IP-10 and IL-8 expressions via hindering NF-κB (p65) and RNA polymerase II from occupancy at the corresponding promoter sites.

Although there are some similarities with the previous published work [13], this study was designed to confirm whether a direct contact of monocytes or alveolar macrophages with Mycobacteria might directly induce NKRF without other immunological modifications as like in vivo. Other immune cells, especially lymphocytes may have primed circulating monocytes or alveolar macrophages even retrieved from normal subjects in this country where TB is highly prevalent and latent TB is difficult to be excluded. Thus, the mechanisms explored in primary cell lines might provide more insight into the up-regulation of NKRF and its interaction with NF-κB in regulation of IP-10 and IL-8 release.

In pulmonary tuberculosis, the encounter between MTb and the innate immune system induces a complicated and sophisticated series of host responses. The next long-term phase of the encounter is played by the activation of the adaptive immune system. An important first step is to recruit intravascular immune cells to the proximity of the infective focus and prepare them for extravasation. In pulmonary tuberculosis, the local generation of chemokines and immune regulation are responsible for the recruitment of leukocytes to the site of inflammation and injured site [29]. The increased induction of C-X-C chemokines, IP-10 in MTb infection [9],[13] attracts Th1-, Tc1-activated lymphocytes and NK cells through CXCR3 [10]-[12]. IP-10 also can play a role in generation and function of effector T cells by promoting antigen-specific proliferation and IFN-γ secretion [12],[30],[31]. IL-8 is involved in monocyte, lymphocyte, and neutrophil recruitment [32],[33], and implicated in granuloma formation and maintenance in TB [21],[34],[35]. The present study demonstrated that direct contacts with MTb induced mononuclear cells to increase synthesis and release of IP-10 and IL-8, indicating IP-10 and IL-8 are very important in the innate immune response and bridges to the adaptive cellular response.

NKRF plays a dual role in IL-1-induced IL-8 transcription [27] and the binding to DNA specifically abolishes the transcriptional activity of the bordering NF-κB-binding sites by a noncompeting, distance and position-independent mechanism [26]. In our previous study in human airway smooth muscle cells, NKRF inhibited neutrophil elastase induced NF-κB transactivating activity or directly suppressed the promoter site to modulate IL-8 synthesis and protein release [36]. In alveolar macrophages and peripheral blood mononuclear cells of pulmonary TB patients, knockdown of NKRF significantly increased IP-10 and IL-8 release [13]. These results suggest NKRF may serve as an endogenous repressor in IP-10 and IL-8 synthesis and release. However, NKRF was found up-regulated to repress IP-10 and IL-8 release only in pulmonary TB patients with high bacterial load [13]. In this study, we have shown H. TB dose-dependently increased NKRF expression in THP-1 cells, significantly at concentrations of H. TB more than 5 μg/ml, suggesting the induction of NKRF synthesis might be directly related to exposure to TB bacillus itself or its components.

MTb and its components also have been reported to cause a constitutive degradation of IκB-α, leading to NF-κB activation in monocytes from TB patients [37]. TransAM assay in the present study showed H. TB exposure induced activation of a variety of NF-κB subunits, predominantly p65. A specific NF-κB inhibitor Helenalin suppressed H. TB induced IP-10 and IL-8 release, suggesting both IP-10 and IL-8 release were mainly mediated via NF-κB activation, especially p65 subunit. Our previous study has indicated that NF-κB activation induces NKRF synthesis through NF-κB subunit p65 binding to the NKRF promoter to transcriptionally activate NKRF mRNA synthesis [36]. Thus, a direct contact with MTb induces NF-κB activation leading to up-regulation of IP-10 and IL-8 synthesis and release in mononuclear cells. If bacterial load is high enough, NF-κB activation may also induce a concomitant increase in NKRF that represses the synthesis and release of IP-10 and IL-8.

A further study to investigate whether H. TB induced up-regulation of NKRF is mediated through NF-κB is hindered by a concomitant NF-κB activation-increased oxidative stress induced by H. TB stimulation. The high levels of oxidative stress degrade NKRF as we have reported in monocytes of COPD patients [38]. Thus, modification of NF-κB activation by pharmacological inhibitors or by gene knock down or over-expression may concomitantly influence the levels of intracellular oxidants, leading to a difficulty in precisely representing the causal-effect on NKRF synthesis in this experimental model. In addition, we have also found distinct subunits of NF-κB are activated by different concentrations of H. TB. Whether distinct NF-κB subunits mediate differential effects of H. TB on NKRF expression is currently under study.

Our data display a great difference of increased activities of p65 and NKRF to promoter of IP-10 and IL-8, *i.e*. NF-κB p65 expression was 2-fold for IP-10 and 15-fold for IL-8. The possible explanation is that the IL-8 promoter is predominantly activated by the induction of NF-κB complex containing p65, though either AP-1 or C/EBP-β may also play supporting roles [39],[40]. However, IP-10 promoter is predominantly activated by an interferon-stimulated response element, although there is also an existent NF-κB responsive site that binds a p65 homodimer. IP-10 gene activation is less dependent on NF-κB p65, but only in maximal IP-10 expression, cooperation between two sites is required [41]. Thus, the binding activities of NF-κB to promoter of IP-10 might be much lower than those to IL-8 promoter.

Our ChIP assay used the same primer pairs (Table 2) to amplify a region around the NRE site in the promoters of IP-10 and IL-8. There was an increase in NKRF occupancy at IP-10 and IL-8 promoter sites in the H. TB treated group, suggesting an inhibition at the transcriptional initiation. RNA interference and plasmid overexpression were performed to explore a link between NKRF expression and it’s repressive effect on IP-10 and IL-8 synthesis. In THP-1 cells, knockdown of NKRF augmented H. TB induced IP-10 and IL-8 release, but overexpression of NKRF attenuated the responses. Furthermore, in ChIP assay we found that NKRF overexpression restrained p65 binding and RNA polymerase II recruitment to IP-10 and IL-8 promoter sites. However, NKRF overexpression did suppress the baseline IP-10 and IL-8 release, and H. TB still induced IP-10 and IL-8 release from THP-1 cells. Therefore, we suggest NF-κB might be also involved in the basal release of IP-10 and IL-8. H. TB induced IP-10 and IL-8 release is not completely inhibited by NKRF. H. TB may act through NF-κB-independent pathways, such as AP-1 or C/EBP-β or interferon-stimulated response elements to induce IL-8, or IP-10 release. Thus, NKRF in THP-1 cells may serve as an endogenous repressor to prevent robust increase in H. TB induced IP-10 and IL-8 release by interference with NF-κB transcriptional activity.

## Conclusions

The results of this in vitro study are consistent with the findings of our previous study that NKRF up-regulated expression in alveolar macrophages and peripheral blood monocytes of active pulmonary TB patients represses IP-10 and IL-8 synthesis and release. This study further delineates the underlying mechanisms that a direct contact with MTb induces NKRF synthesis and nuclear translocation, binding to NRE in the promoter sites of IP-10 and IL-8. Over there, NKRF interferes with NF-κB (p65) binding and RNA polymerase II recruitment, leading to a repressive effect on IP-10 and IL-8 synthesis.

## Abbreviations

ChIP: Chromatin immunoprecipitation

ECL: Enhanced chemiluminescence

FCS: Fetal calf serum

H. TB: heated mycobacterium tuberculosis

IL-8: interleukin-8

IP-10: gamma interferon induced protein 10 kd

MTb: Mycobacteria Tuberculosis

NF-κB: Nuclear factor kappa B

NKRF: Nuclear factor kappa B repressing factor

NRE: negative regulatory element

PBS: Phosphate-buffered saline

q-PCR: Quantitative polymerase chain reaction

RT-PCR: Reverse transcription-polymerase chain reaction

siRNA: Small interfering RNA

TB: Tuberculosis

## Competing interests

The authors declare that they have no competing interests related to this work.

## Authors’ contributions

KHH, CHW, HPK and CHL designed and written this study. KHH performed this study. All authors read and approved the final manuscript.
